# Cost-effective optimized method to process 3D tumoral spheroids in microwell arrays for immunohistochemistry analysis

**DOI:** 10.25122/jml-2024-0267

**Published:** 2024-06

**Authors:** Mircea Bogdan Matei, Carmen Letitia Marinescu, Christien Oktaviani Matei, Alex-Sebastian Pînzariu, Leon Zăgrean, Mihaela Georgeta Moisescu

**Affiliations:** 1 Physiology and Neuroscience Department, Carol Davila University of Medicine and Pharmacy, Bucharest, Romania; 2 Biophysics and Cellular Biotechnology Department, Carol Davila University of Medicine and Pharmacy, Bucharest, Romania; 3 Pathology Department, Sante Medical Clinic, Bucharest, Romania; 4 Excellence Center for Research in Biophysics and Cellular Biotechnology, Carol Davila University of Medicine and Pharmacy, Bucharest, Romania

**Keywords:** 3D spheroid culture, microwell array, immunohistochemistry, pathology, glioblastoma, breast adenocarcinoma

## Abstract

This study presents an improved method for obtaining spheroids microwell arrays for histological processing and analysis, focusing on glioblastoma (U87 MG) and breast adenocarcinoma (MCF-7) tumor models. By transitioning from traditional 2D cell cultures to 3D systems, this approach overcomes the limitations of 2D cultures by more accurately replicating the tumor microenvironment. The method consists of producing homotypic and heterotypic spheroids using low-adherence agarose-coated wells, embedding these spheroids in agarose microwell arrays, and conducting immunohistochemistry (IHC) to analyze cellular and molecular profiles. Morphological analyses were performed using OrganoSeg software, and IHC staining confirmed marker expressions consistent with respective tumor types. The study details the workflow from 2D cell culture to IHC analysis, including agarose well coating, spheroid embedding, and IHC staining for markers such as EMA, p53, Ki-67, ER, PR, and HER2. Results demonstrated compact, round U87 MG spheroids and fibroblast-stabilized MCF-7 spheroids, with both types exhibiting specific marker expressions. This innovative approach significantly enhances the efficiency of producing and analyzing large volumes of spheroids, making it both quick and cost-effective. It offers a robust drug screening and cancer research platform, maintaining spheroid traceability even in bulk workflow conditions. Furthermore, this methodology supports advances in personalized medicine by providing a more physiologically relevant model than 2D cultures, which is crucial for investigating tumor behavior and therapeutic responses through IHC.

## INTRODUCTION

The evolution of cell culture techniques from two-dimensional (2D) to three-dimensional (3D) models represents a paradigm shift in cancer research, profoundly impacting our capabilities to understand the disease and to develop new therapies. While 2D cultures have played an important role in the initial exploration of cellular behavior and drug testing, their limitations in accurately simulating the complex architecture and microenvironment of tumors have led to the adoption of 3D culture systems [1].

It is considered that in 2D culture systems, the planar growth leads to artificial cytoskeletal polarization, causing diminished cell-to-cell and cell-to-matrix interactions, leading to altered cell morphology, proliferation rates, and gene expression profiles [2]. They also fail to properly form microenvironmental niches, which is vital for portraying tumoral heterogeneity observed in vivo [3]. Hence, results from 2D cultures often do not translate well to clinical settings, prompting the search for more representative models.

**Figure 1:**
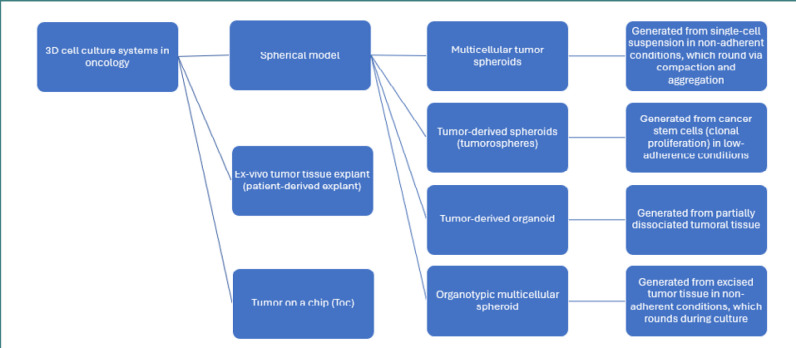
Main categories of 3D culture models

The introduction of 3D cell culture techniques marked a significant advance, offering models that better mimic the physical and biochemical cues of the tumor microenvironment [4]. 3D cultures allow cells to grow in all directions, forming structures that exhibit cellular heterogeneity and complex cell-to-cell and cell-to-matrix interactions while exposed to nutrient and oxygen gradients. These conditions lead to a more physiologically relevant behavior, including more accurate responses to therapeutic agents [5,6]. The main categories of 3D systems used in oncological research are represented in Figure 1.

Patient-derived explants and tumor-on-a-chip systems represent more advanced categories of 3D tumor models due to their added complexity, though their application is limited by the technical challenges associated with their production. Patient-derived explants involve culturing fresh tumor tissues extracted directly from patients, preserving various cell types and the original tumor micro-architecture. This method maintains critical features of the tumor microenvironment, such as cell-to-cell communication, tumor-stroma interactions, and heterogeneity, providing a valuable tool for personalized medicine approaches [7]. The tumor-on-a-chip engineering integrates microfluidics with 3D cell culture and provides appropriate models for vasculature interfaces and tissue dynamics. Tumors-on-a-chip can also explore tumor growth, metastasis, and the interaction between tissue types. They also allow for the precise control of microenvironmental conditions, such as nutrient flow and mechanical forces, offering insights into how these factors influence cancer progression and response to treatment [8].

The spheroidal approach of 3D culture represents the most widely applied method of culturing and includes techniques that differ in terms of tumor cell source, cell handling, culture surface, and time required to form a 3D structure (Figure 1).

**Figure 2:**
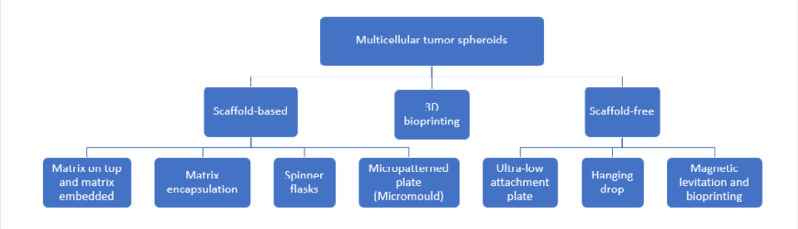
Main techniques used for spheroids formation

The techniques for spheroid formation may be classified into three main categories: scaffold-based, scaffold-free, and 3D bioprinting (Figure 2). The scaffold-based techniques include seeding cells into or onto three-dimensional acellular scaffolds made of various biocompatible materials that mimic specific tissue architecture. The scaffolds can be produced from either non-biodegradable material (agarose, alginate, ceramics, polystyrene, etc.) [9] or biodegradable materials (collagen, polyglycolic acid, polylactic acid, gelatin, etc.) [10]. Generation and tailoring of scaffolds use various processes, including electrospinning, cryogenic electrospinning, gas foaming, and solvent casting with particulate leaching [11]. In scaffold-free techniques, key methods promoting spheroid formation include hanging drop, coating surfaces with agarose or hydrogel, using low adherence plates, employing magnetic levitation, rotating wall vessel bioreactors, and integrating microfluidic devices [2,12]. Both scaffold-based and scaffold-free approaches encounter challenges in achieving native-like cell density, vascularity, and the capability for tissue remodeling.

3D bioprinting is a convergence of spheroid production and bioprinting and enables the precise deposition of spheroids into hydrogels to construct tissue-like structures [13,14]. This technique allows for the creation of complex, multicellular tumor models that include not just cancer cells but also stromal and immune cells, accurately recreating the tumor microenvironment as well. 3D bioprinted models can be customized to replicate specific tumor types and microenvironments, offering a powerful platform for drug screening and disease modeling.

Furthermore, in the context of ongoing regulatory updates at the Food and Drug Administration, cellular 3D and computer models are increasingly prioritized as alternatives to animal testing. These models align with the 3Rs of animal experiments—Replacement, Reduction, and Refinement—to assess the safety and effectiveness of drugs [15,16]. In drug development, 3D models have improved the predictive accuracy of preclinical testing, reducing the failure rate of drugs in clinical trials by better mimicking how cancer cells respond to treatments in personalized medicine approaches.

Considering the increasing interest in developing 3D cell culture models, the techniques to obtain such models are getting better in terms of outcome and resource exploitation. Moreover, the methods used for the complex characterization of these models (e.g., antibody multiplexing) must keep pace with this fast development. To evaluate various research targets based on radial position within a spheroid, obtaining high-magnification images is critical to ensure accurate quantitative representations. However, 3D imaging involves a trade-off between maximal numerical aperture (essential for Z-resolution) and working distance. A larger numerical aperture results in shorter working distances, limiting imaging depth. Therefore, selecting an optimal method for volume rendering depends on the required level of detail. Despite advances allowing deeper tissue penetration through clearing techniques, challenges such as antibody penetration and fluorophore spectrum overlap remain significant obstacles for in situ imaging studies [17]. Although there is a continuously growing body of methods to analyze 3D cultures, immunohistochemistry (IHC) remains the gold standard for the histological evaluation of the interactions between cells and stroma (especially when the spheroid’s core sections are of interest) [18].

This paper proposes a method for embedding spheroids in agarose microwell arrays specifically designed to facilitate subsequent IHC processing. This technique enables simultaneous sectioning of an entire batch of 3D cultured spheroids, with each histological section providing an opportunity for cellular and molecular profiling of designated regions. The feasibility of the procedure was tested on spheroids derived from U87 MG glioblastoma and MCF-7 adenocarcinoma cells. Several IHC tests were performed on 48 spheroid grids per glass slide while maintaining annotations of their well locations.

## MATERIAL AND METHODS

### Spheroid formation

#### 2D cell culturing

U87 MG (human primary glioblastoma, HTB-14, ATCC), MCF-7 (human breast adenocarcinoma, HTB-22, ATCC), and Hs27 (human fibroblast, CRL-1634, ATCC) cell lines (at a low passage number) were propagated in 2D culture according to their specific protocol and under standard growth conditions (5% CO_2_, 37^o^C, humid atmosphere). The growth medium was used according to cell line specifications as follows. For MCF-7 and Hs27: EMEM (Eagle's Minimum Essential Medium, 30-2003, ATCC) enriched with 10 µg/mL human insulin (Novorapid Flex Pen, 100 IU/mL, Novo Nordisk) and 10% fetal bovine serum (F7524, Sigma-Aldrich), adapted after one study [19]. For U87 MG, the following culture medium was used: EMEM (Eagle’s Minimum Essential Medium, 30-2003, ATCC) enriched with 10% fetal bovine serum.

#### Agarose well coating

A 1% sterile agarose solution (A9539, Sigma) was prepared by dissolving 0.25 g of agarose in 25 mL of double-distilled water and heating it to 100°C for 1 minute. Low-adherent 96-well round bottom plates (650160, Cellstar) were then coated with this solution, which was maintained above its melting point. For each well, 150 µL of the agarose solution was dispensed and evenly distributed to form a thin, low-adherent agarose film on the bottom of the wells. The plates were then sealed with parafilm and stored at 4°C.

#### 3D cell culturing

For 3D culturing, cells from 2D cultures were harvested using Trypsin-EDTA (T4174, Sigma) and then counted using a BioRad TD10. Cell suspensions were seeded into each agarose-coated well containing 200 µL of medium at a density of 2 x 10^4^ per well using a liquid overlay technique. The cultures were monitored for medium depletion, and, if necessary, 100µL of medium was exchanged by gentle pipetting in/out on the well wall. Two categories of spheroids were prepared: homotypic (for U87 MG and MCF-7) and heterotypic (co-cultures of MCF-7/Hs27 in ratios of 3/1 or 1/1). For longer growth periods, such as the 14-day culture of homotypic MCF-7 spheroids, the medium was refreshed on days 5 and 10 by replacing 100 µL with fresh medium. The growth medium was removed on the day of embedding, and the spheroids were fixed using 4% paraformaldehyde for 24 hours.

### Spheroid morphological analysis

#### Image acquisition

Spheroid images were acquired with an inverted brightfield microscope (Axiovert 200 equipped with AxioCam MRm controlled by AxioVision 6.9, Zeiss) using a 4x objective. This setup allows the entire well to be viewed clearly, with each image pixel corresponding to 3.448 µm. Images were acquired from each well on different days. The conditions in which the images were obtained were identical (illumination parameters, objective, exposure time, etc.). Images were stored as .tiff files and named according to well origin and date.

#### Image segmentation and parameters for analysis

The OrganoSeg software (a digital image segmentation plugin developed under MATLAB) [20] was used to profile 3D cultures over time. The software was preferred due to its capability to address challenges like a large number of images, artifact shapes, discrimination from cell debris, nonuniform blur, and spherical aberration from low-magnification imaging that could alter the analysis results.

Segmentation consisted of obtaining an optimal contour delineation of the spheroid image by dynamically varying the three parameters: Intensity (Otsu) threshold, Window size, and Size threshold, as defined by another study [20]. The following parameter ranges were used for segmentation: Intensity (Otsu) threshold = 0.183–1, Max-window size = 100–370 pixels, Size threshold = 148–3130 pixels. On segmented images, various morphological and statistical parameters may be computed (e.g., axes, area, perimeter, kurtosis, skewness, correlation).

In this study, the growth of spheroids was monitored by measuring the morphological parameter volume over time, computed according to the equation:

*V* = 0.5 • *L* • *l* ^2^

where *L* is the long axis and *l* is the short axis of the spheroid [21].

As OrganoSeg exports data as one image–one worksheet, an Ablebits Microsoft Excel extension (Office Data Apps) was used to merge individual worksheets into one [22].

For further analysis, only spheroids satisfying the following morphological criteria were selected: (1) a minimum *l* ≥ 200 µm, (2) the presence of a single spheroid per well, and (3) a sphericity index (*SI*) > 0.6 (where $SI=\sqrt{L/l}$).

### Agarose microwell array

#### Mold design and printing

**Figure 3:**
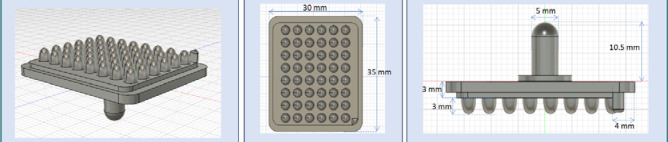
Schematics of the mold used for obtaining the agarose microwell array

A mold was designed in Fusion360 and printed from poly-lactic acid using a filament printer (Snapmaker 2.0). The mold features 48 rounded parabolic pegs, each 3 mm in height and 2 mm in maximum diameter, arranged in 6 columns and 8 rows. (Figure 3). The design also includes a handlebar on the opposite side of the pegs to facilitate handling. Prior to use, the mold was sprayed with silicone to ensure easy release from the agarose without adherence.

#### Agarose microwell array fabrication

**Figure 4:**
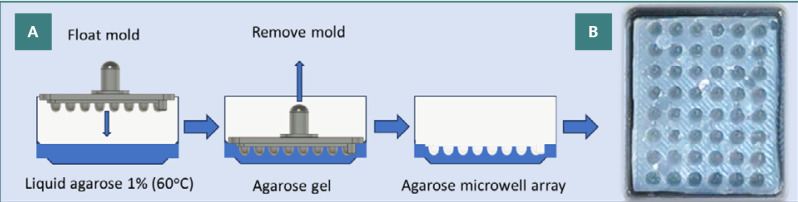
Fabrication and visualization of agarose microwell arrays. A, Workflow for obtaining the agarose microwell array; B, Image of the resulting agarose microwell array.

Melted agarose solution was pipetted in a tray (with appropriate dimensions for the mold to fit in). The mold was then allowed to float on the surface of the liquid agarose (Figure 4A). To eliminate any trapped air bubbles, the handlebar was gently pressed and tapped. The agarose solution was allowed to gel for 5 min at 4^o^C and 1 min at -18^o^C. Then, the mold was gently removed from the tray, and an agarose 48 microwell array was formed (Figure 4B). Each microwell accommodates approximately 50 µL of liquid.

### Spheroids embedding

The fixed spheroids were gently transferred (with approximately 50 µL of paraformaldehyde) from the 96 well plates to two agarose microwell arrays (totaling 96 microwells). For the transfer, 200 µL tips were adjusted by cutting, thus allowing for spheroids to pass without disruptions. Afterward, the agarose microwell array was subjected to centrifugation at 100×g for 1 min to provide an even layering of the spheroids into the microwells. The array was then covered with a layer of approximately 1.5 mm height of 1% low melting agarose (A4108, Sigma). The agarose displaces thus the paraformaldehyde solution and air bubble trapping is avoided. The microarrays containing the sealed embedded spheroids were stored in paraformaldehyde before IHC.

### Immunohistochemistry

#### Sample preparation for IHC

The sealed microwell arrays were placed in histological cassettes and processed using a tissue processor (Epredia Excelsior AS). Briefly, they were dehydrated in a series of alcohol solutions with increasing concentrations (50%, 70%, 90%) followed by four baths of absolute methanol, 1 h each. Afterward, the samples were cleared in xylene (three baths, 1 h each) and infiltrated with melted paraffin (two baths, 2 h each, under vacuum). Further, the samples were subjected to paraffin embedding (Leica HistoCore – Arcadia H). Then, the samples were cut into 3 µm thick slices (Leica HistoCore Biocut) and placed on glass slides to dry.

#### Sample IHC staining

Slides were stained for EMA, p53, Ki-67 (for U87 MG spheroids) and for ER, PR, HER2, and Ki-67 (for MCF-7 spheroids) using an automated staining machine (BenchMark GX IHC/ISH). Roche Ventana monoclonal antibodies were used following the manufacturer protocols:

i/ for U87 MG: Anti-EMA (E29) Mouse Monoclonal Primary Antibody (790-4463), Anti-p53 (Bp53-11) (760-2542) Mouse Monoclonal Primary Antibody, Anti-Ki-67 (30-9) Rabbit Monoclonal Antibody (790-4286);

ii/ for MCF-7: Anti-Estrogen Receptor (ER) (SP1) Rabbit Monoclonal Primary Antibody (790-4324), Anti-Progesterone Receptor (PR) (1E2) Rabbit Monoclonal (IgG) Primary Antibody (790-2223), Anti-HER2/neu (4B5) Rabbit Monoclonal Primary Antibody (790-2991), Anti-Ki-67 (30-9) Rabbit Monoclonal Antibody (790-4286).

The presence of the spheroids in each microwell and their integrity were confirmed by standard hematoxylin and eosin (H&E) staining (Leica Autostainer XL).

## RESULTS

**Figure 5:**
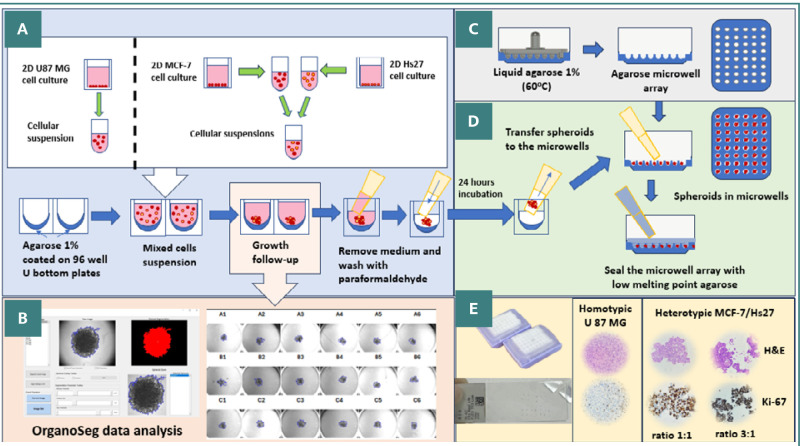
Comprehensive workflow for spheroid preparation and analysis. A, Generation of homotypic and heterotypic spheroids from U87 MG and MCF-7 cell suspensions, followed by their growth on a low-adherence agarose surface; B, Microscopic image acquisition of spheroids and their morphological evaluation with OrganoSeg; C, Production of agarose microwell arrays; D, Transfer and embedding of spheroids into the agarose microwell array; E, H&E and IHC staining and analysis of spheroids.

Figure 5 (A-E) presents the main workflow steps from 2D cell culturing to IHC analysis of homotypic or heterotypic spheroids.

The growth rates, compactness, and manipulation stability of spheroids varied among different cell types. By monitoring the spheroids under a microscope and using OrganoSeg analysis, researchers can determine the optimal day to collect spheroids for embedding and subsequent IHC evaluation.

**Figure 6:**
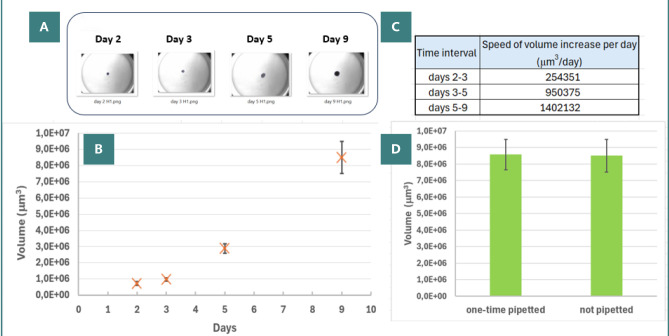
Homotypic spheroids from U87 MG cell line. A, Sequential images of the same spheroid taken on days 2, 3, 5, and 9 post-seeding; B, Changes in spheroid volume over time, illustrating growth from the day of cell seeding; C, Analysis of growth rates, depicted as the speed of volume increase per day, during different stages of spheroid formation; D, Stability to manipulation as reflected in volumes at day 9 for two categories of spheroids: one-time pipetted versus not pipetted.

Homotypic spheroids obtained from the U87 MG cell line were compact and round (Figure 6A). The volume of spheroids increased over the monitoring period (Figure 6B), displaying varying growth rates from the time of cell seeding (Figure 6C). To assess the impact of mechanical stress on morphological parameters and integrity, we conducted a test to compare the volume changes in spheroids that were undisturbed since seeding with those that had been pipetted once within the same well (Figure 6D, volume values corresponding to day 9).

**Figure 7:**
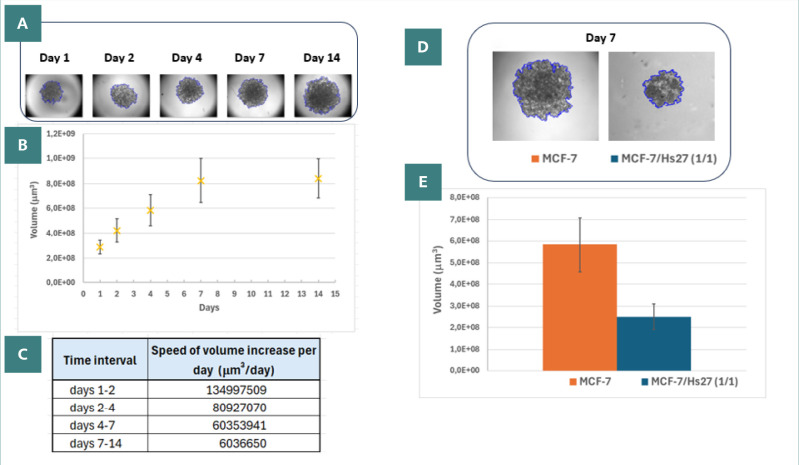
MCF-7 spheroids. A, Sequential images of a homotypic MCF-7 spheroid taken at days 1, 2, 4, 7, and 14 post-seeding, with edges highlighted by OrganoSeg in blue; B, Graph showing the changes in the volume of homotypic spheroids over time from the day of seeding; C, Evaluation of spheroids growth as the speed of volume increase per day, for different periods during the formation of homotypic type; D, Images at day 7 of a homotypic and a heterotypic spheroid; E, Comparison in volumes at day 7 between homotypic and heterotypic spheroids (MCF-7/Hs27, 1/1).

The MCF-7 spheroids had a different behavior than the U87 MG ones. The MCF-7 homotypic spheroids were loose and had irregular shapes (Figure 7A), and their integrity was easily lost at the moment of pipetting (data not shown). The volume of homotypic MCF-7 spheroids increased during the days of monitoring, reaching a plateau in growth at day 14 (Figure 7B). This plateau was confirmed by the decrease in the speed of growth for the second week of growth (Figure 7C). The addition of human fibroblast Hs27 to MCF-7 spheroids (MCF-7/Hs27 ratio 1/1 or 3/1) improved their stability, allowing further manipulation. The Hs27 fibroblasts compacted the MCF-7 epithelial spheroids (Figure 7D), reflected by the decrease in volume in the case of heterotypic spheroids (Figure 7E).

**Figure 8:**
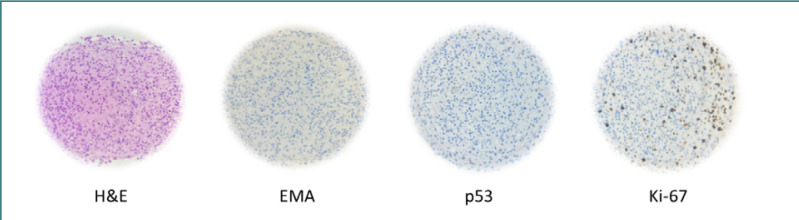
Histological and immunohistochemical staining of U87 MG homotypic spheroid

The H&E staining of U87 MG spheroids (Figure 8) showed malignant tumor proliferation, consisting of anaplastic cells with intense-eosinophilic cytoplasm (focally vacuolated). Hyperchromic, vesicular, and pleomorphic nuclei with prominent nucleoli and rare mitotic figures were also present. In spheroids larger than 400 µm in diameter, H&E staining revealed a central necrosis area, which appears as an anucleate eosinophilic amorphous region, as shown in Figure 8 – H&E.

The epithelial membrane antigen (EMA) evaluation was negative, with no EMA dots or ring-like structures observed per high-power field (Figure 8 – EMA). A significant loss of p53 expression was noted, with nuclear expression in less than 5% of the spheroid cells (Figure 8 – p53). In contrast, intense nuclear expression of Ki-67 in approximately 20-25% of the spheroid’s cells (Figure 8 – Ki-67) indicated high proliferation activity. These findings confirm the presence of a glioblastoma-type tumor characterized by diffused glioma and anaplastic features.

**Figure 9:**
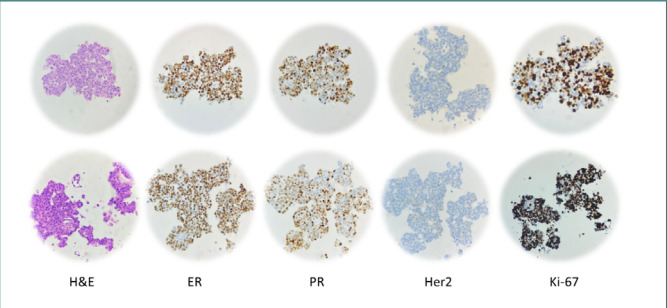
Histological and IHC images of heterotypic spheroids: MCF-7/Hs27, ratio 1/1 (top row) and MCF-7/Hs27, ratio 3/1 (bottom row)

The H&E staining of heterotypic MCF-7/Hs27 spheroids, with seeding ratios of 1/1 and 3/1 (Figure 9, top and bottom, respectively), showed malignant proliferation characterized by medium-sized, oval-shaped cellular placards with distinct boundaries. The cells exhibited abundant eosinophilic cytoplasm and large, hyperchromic, or vesicular round-oval nuclei with moderate pleomorphism, prominent nucleoli, and rare, atypical mitoses.

IHC staining showed that approximately 70% of cells expressed nuclear ER, and about 60% showed PR nuclear expression (similar results were obtained for both categories of spheroids). The IHC staining for HER2 was negative in both categories since incomplete and barely perceptible membrane expression in <10% of spheroid cells were visible.

Ki-67 staining revealed nuclear expression in about 70% of cells in spheroids with a 1:1 seeding ratio (Figure 9 Ki-67, top) and in more than 80% of cells in the other group (Figure 9 Ki-67, bottom). Thus, both heterotypic spheroids were considered Ki-67 positive since more than 20% of their cells were stained. These results confirmed the presence of invasive breast carcinoma, indicative of malignant ductal epithelium proliferation in the mammary gland (Luminal B-like HER2 negative).

## DISCUSSION

Spheroids from U87 MG and MCF-7 cell lines were successfully cultured in wells coated with agarose using a liquid overlay technique. This approach prevented cell adhesion to the culture surface and subsequent evolution towards 2D culture. Agarose was chosen for its biocompatibility, nutrient permeability, non-adhesive properties, and cost-effectiveness. Embedding the spheroids in an agarose microwell array facilitated easy access to immunohistochemistry procedures.

U87 MG cells produced spheroids compact enough for embedding as homotypic spheroids. In contrast, MCF-7 cells, after 14 days of growth, did not achieve sufficient mechanical resistance to pipetting to permit embedding. For MCF-7, the addition of Hs27 fibroblast cells was necessary to enhance the spheroids’ structural integrity. It is known that, in vivo, aggressive glioblastoma tumors develop high-density stroma (microenvironment) [23]. In the case of breast adenocarcinoma, the epithelial origin of the tumor exhibits a less dense microenvironment [24]. The fibroblasts are known to be the main contributor to the microenvironment stiffness [25].

Moreover, adding Hs27 fibroblasts facilitated the compactness required for embedding and reduced the time needed to develop transferable spheroids, eliminating the need for frequent growth medium renewal. Immunohistochemistry staining for both categories of MCF-7 spheroids was consistent with expression patterns needed to classify breast tumor types [26,27]. A necrotic core was observed in all spheroids larger than 400 µm in diameter, regardless of cell type, due to limited oxygen diffusion in spheroids exceeding 150–200 µm in diameter [28].

The agarose microwell array facilitates the rapid collection and room temperature storage of large spheroid libraries produced in vitro. This capability is particularly beneficial for separating workflow stages, such as microwell array production and subsequent sample staining and analysis. This feature proves very useful when a large set of different experimental conditions requires analysis on the same slide or in the same experimental run, considering that a single array contains 48 spheroids, with up to 50 slices for only 20% of the spheroid core if the sample has more than 400 µm diameter. Thus, the number of biological enquiries (addressed using the same set of slices) can increase to tens and even hundreds.

Similarly, there is a recognized (pressing) need to automate spheroid transfer to the microwell arrays, reducing the variability of the spheroid dimensions artifacts. Moreover, if spheroids of different sizes are embedded in the same microwell array, a mismatch between the z sections may occur. Addressing the final gap in workflow automation requires speeding up the image analysis process. This enhancement would involve microscope manufacturers (such as providers of inverted microscopes and slide scanners) offering options to automatically capture annotated images suitable for batch analysis in software like OrganoSeg or ImageJ.

Several challenges addressed by this method include:

- The in/out slow pipetting of melted agarose maintained a pronounced U-shape of the wells (a thin layer being thus obtained) without decreasing the volume available for the growth medium.
- The pipetting of spheroids was done using cut tips, allowing the spheroids to pass without any mechanical stress.
- The types of agaroses used for the microwell array (production and sealing) were chosen to resist the processing for IHC (dehydration, paraffin inclusion, microtome sectioning, etc.).
- For sealing the spheroids, low melting point agarose was carefully layered on the paraformaldehyde-filled wells. The higher density of agarose displaces the paraformaldehyde, effectively preventing the formation of air bubbles.

Confocal microscopy for in vivo 3D evaluation of spheroids would also be a straightforward method to address cellular and environmental cues. However, when ex vivo objects up to 1 mm are evaluated, two-photon confocal microscopy with clearing methods must be used [29], a much more sophisticated technique compared to various automated IHC workflows already in clinical use. For cancer research, including the evaluation of cellular phenotypes and safety/efficacy screenings, pathologists may prefer the proposed method for handling histological samples as small as 1 mm or less.

Further work could focus on the hydrophobic layer (agarose coating) of the culture wells, where various components can be added, such as fluorescent particles (rhodamine) or components for metabolic assays (such as lipids if lipases are targeted). Additionally, research could be tailored based on spheroid size; studies might focus on spheroid genesis during the first three days of culture for diameters less than 250 µm, or on histopathological studies of hypoxia for spheroids larger than 300 µm [30].

According to previous experimental data [31], spheroid models can develop subpopulations of cells within the middle quiescent layer. These cells may exhibit higher resistance to treatment or increased survival capacity in specific pathophysiological niches.This observation warrants further evaluation, and if confirmed, it could significantly impact future experimental work, including developing new, targeted drugs.

## CONCLUSION

Innovative technological improvements have reinstated the increasing interest in various spheroid cultures as pertinent models for drug screening or histological microenvironment-related studies. A simple, well-defined workflow was optimized to obtain high output in terms of the number of spheroids over short periods to obtain homotypic (U87 MG) and heterotypic (MCF-7/Hs27) types of spheroids dedicated to IHC analysis. The spheroid's integrity and growth rates were evaluated by morphological parameters analysis carried out semi-automatically using dedicated software for large pools of images. The spheroids batches were embedded in an agarose-based microwell array and were sectioned and stained with cell type-specific markers to reveal protein expression patterns while keeping the spheroid traceability in conditions of bulk workflow.

## Data Availability

Further data is available from the corresponding author on reasonable request.

## Personal thanks

The authors thank Professor Tudor Savopol from the Excellence Center for Research in Biophysics and Cellular Biotechnology of the Carol Davila University of Medicine and Pharmacy, Bucharest, for critically evaluating the manuscript. The authors thank Lecturer PhD Ioan Tivig from the Biophysics and Cellular Biotechnology Dept. of the Carol Davila University of Medicine and Pharmacy, Bucharest, for valuable help designing and printing the mold.

## References

[1] Promega (2023). Reproducible Drug Screening Assays Using Single Organoids. https://www.promega.ro/resources/pubhub/2023/reproducible-drug-screening-assays-using-single-organoids/.

[2] Santo VE, Estrada MF, Rebelo SP, Abreu S, Silva I, Pinto C, Veloso SC, Serra AT, Boghaert E, Alves PM, Brito C (2016). Adaptable stirred-tank culture strategies for large scale production of multicellular spheroid-based tumor cell models. J Biotechnol.

[3] Seguin L, Desgrosellier JS, Weis SM, Cheresh DA (2015). Integrins and cancer: regulators of cancer stemness, metastasis, and drug resistance. Trends Cell Biol.

[4] Friedrich J, Ebner R, Kunz-Schughart LA (2007). Experimental anti-tumor therapy in 3-D: spheroids--old hat or new challenge?. Int J Radiat Biol.

[5] Ivanov DP, Grabowska AM (2017). Spheroid arrays for high-throughput single-cell analysis of spatial patterns and biomarker expression in 3D. Sci Rep.

[6] Rama-Esendagli D, Esendagli G, Yilmaz G, Guc D (2014). Spheroid formation and invasion capacity are differentially influenced by co-cultures of fibroblast and macrophage cells in breast cancer. Mol Biol Rep.

[7] Weiswald LB, Bellet D, Dangles-Marie V (2015). Spherical cancer models in tumor biology. Neoplasia.

[8] Neophytou CM, Panagi M, Stylianopoulos T, Papageorgis P (2021). The Role of Tumor Microenvironment in Cancer Metastasis: Molecular Mechanisms and Therapeutic Opportunities. Cancers (Basel).

[9] Nikolova MP, Chavali MS (2019). Recent advances in biomaterials for 3D scaffolds: A review. Bioact Mater.

[10] Reineke B, Paulus I, Löffelsend S, Yu CH, Vinogradov D, Meyer A (2024). On-chip fabrication and in-flow 3D-printing of microgel constructs: from chip to scaffold materials in one integral process. Biofabrication.

[11] Robert Lanza RL, Joseph P, Robert Lanza RL, Joseph P. Vacanti, Anthony Atala (2020). Vacanti, Anthony Atala Principles of Tissue Engineering-Academic Press.

[12] Ryu NE, Lee SH, Park H (2019). Spheroid Culture System Methods and Applications for Mesenchymal Stem Cells. Cells.

[13] Daly AC, Davidson MD, Burdick JA (2021). 3D bioprinting of high cell-density heterogeneous tissue models through spheroid fusion within self-healing hydrogels. Nat Commun.

[14] Banerjee D, Singh YP, Datta P, Ozbolat V, O'Donnell A, Yeo M (2022). Strategies for 3D bioprinting of spheroids: A comprehensive review. Biomaterials.

[15] FDA (2021). Three-Dimensional (3D) Cell Culture (Microphysiological) Platforms as Drug Development Tools.

[16] Ortolano N (2024). Organoids bring drug discovery and development to the culture hood 10x Genomics2023; cited 12th of June. https://www.10xgenomics.com/blog/organoids-bring-drug-discovery-and-development-to-the-culture-hood.

[17] Leary E, Rhee C, Wilks BT, Morgan JR (2018). Quantitative Live-Cell Confocal Imaging of 3D Spheroids in a High-Throughput Format. SLAS Technol.

[18] Ivanov DP, Grabowska AM (2018). In Vitro Tissue Microarrays for Quick and Efficient Spheroid Characterization. SLAS Discov.

[19] Heneweer M, Muusse M, Dingemans M, de Jong PC, van den Berg M, Sanderson JT (2005). Co-culture of primary human mammary fibroblasts and MCF-7 cells as an in vitro breast cancer model. Toxicol Sci.

[20] Borten MA, Bajikar SS, Sasaki N, Clevers H, Janes KA (2018). Automated brightfield morphometry of 3D organoid populations by OrganoSeg. Sci Rep.

[21] Chen W, Wong C, Vosburgh E, Levine AJ, Foran DJ, Xu EY (2014). High-throughput image analysis of tumor spheroids: a user-friendly software application to measure the size of spheroids automatically and accurately. J Vis Exp.

[22] Łomianki P, Office Data Apps Warszawska 109 l5092 (2024). Ablebits Ultimate Suite for Microsoft Excel.

[23] Miroshnikova YA, Mouw JK, Barnes JM, Pickup MW, Lakins JN, Kim Y (2016). Tissue mechanics promote IDH1-dependent HIF1α-tenascin C feedback to regulate glioblastoma aggression. Nat Cell Biol.

[24] Soysal SD, Tzankov A, Muenst SE (2015). Role of the Tumor Microenvironment in Breast Cancer. Pathobiology.

[25] Yakavets I, Francois A, Benoit A, Merlin JL, Bezdetnaya L, Vogin G (2020). Advanced co-culture 3D breast cancer model for investigation of fibrosis induced by external stimuli: optimization study. Sci Rep.

[26] Hofmann S, Cohen-Harazi R, Maizels Y, Koman I (2022). Patient-derived tumor spheroid cultures as a promising tool to assist personalized therapeutic decisions in breast cancer. Transl Cancer Res.

[27] Gnant M, Thomssen C, Harbeck N, St Gallen/Vienna (2015). A Brief Summary of the Consensus Discussion. Breast Care (Basel).

[28] Barisam M, Saidi MS, Kashaninejad N, Nguyen NT (2018). Prediction of Necrotic Core and Hypoxic Zone of Multicellular Spheroids in a Microbioreactor with a U-Shaped Barrier. Micromachines (Basel).

[29] Brenna C, Simioni C, Varano G, Conti I, Costanzi E, Melloni M (2022). Optical tissue clearing associated with 3D imaging: application in preclinical and clinical studies. Histochem Cell Biol.

[30] Pinto B, Henriques AC, Silva PMA, Bousbaa H (2020). Three-Dimensional Spheroids as In Vitro Preclinical Models for Cancer Research. Pharmaceutics.

[31] Friedrich J, Seidel C, Ebner R, Kunz-Schughart LA (2009). Spheroid-based drug screen: considerations and practical approach. Nat Protoc.

